# Supplementary material for: The ULK3 kinase is a determinant of keratinocyte self-renewal and tumorigenesis targeting the arginine methylome
Source: Nat Commun. 2023 Feb 16;14:887. doi: 10.1038/s41467-023-36410-6 (PMC9935893; doi:10.1038/s41467-023-36410-6)
Supplement: Supplementary file 1 — Supplementary Information [file 41467_2023_36410_MOESM1_ESM.pdf]

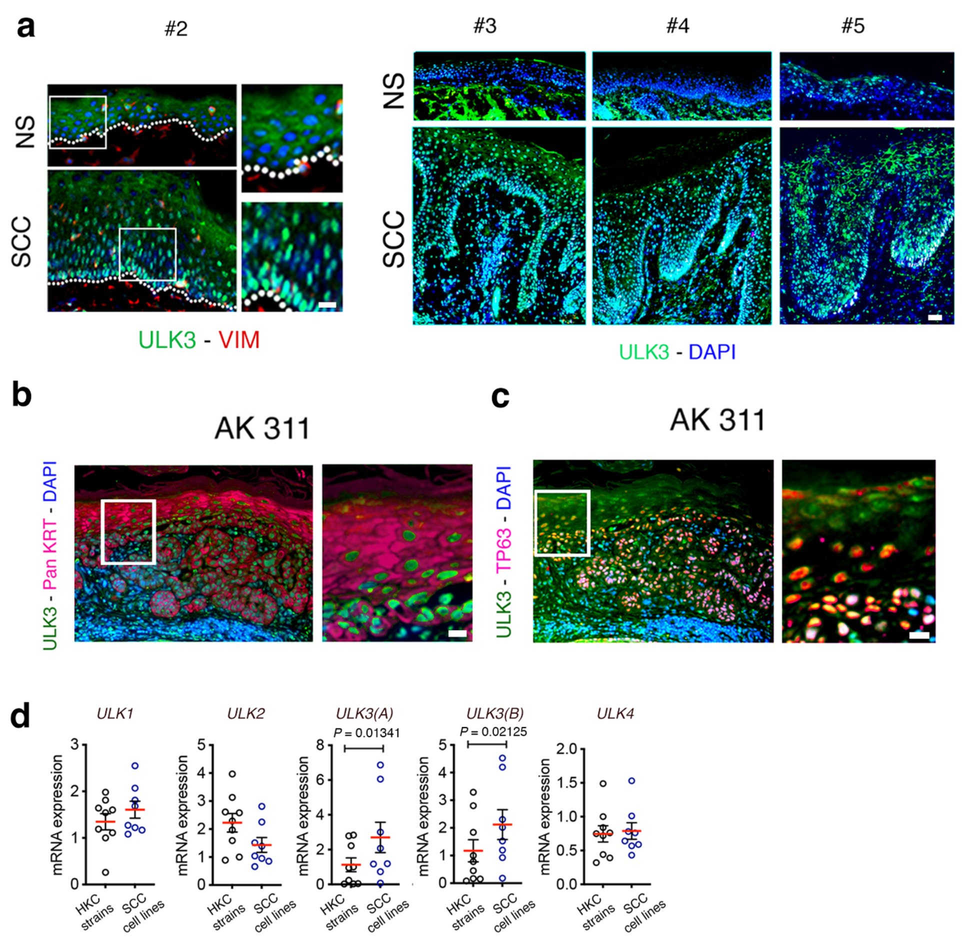

Supplementary Figure 1

a) *ULK3 is upregulated in clinical skin squamous cell carcinoma (SCCs)*. ULK3 (green) and VIMENTIN (magenta) immunofluorescence of a patient-derived skin SCC and normal skin (NS) samples (#2-5), of which ULK3 nuclear localization is quantified in Fig. 1c. DAPI counterstained the nuclei (blue). Low and high magnifications. The white dotted lines show the epithelial-stromal junction. Scale bars 5μM and 100 μM

b) *ULK3 is upregulated in clinical skin actinin keratosis (AKs).* ULK3 (green) and pankeratin (magenta) immunofluorescence of one additional skin AK lesion, quantified in Fig. 1e. Scale Bar 10μM

c) *Co-staining of ULK3 and TP63 in skin AKs.* ULK3 (green) and TP63 (magenta) immunofluorescence of the same skin AK as in panel (b), quantified in Fig. 1f. DAPI stained nuclei (blue). Scale Bar 10μM.

d) *ULK3 is upregulated in SCC cells.* RT-qPCR analysis of the four *ULK* family members expression (ULK1-4), normalized to *36β4*, in each of the nine primary human keratinocytes strains (HKC # GB2-# GB10, black circles) and SCC cell lines (blue circles) used in Fig. 1g. Two couples of oligos, *ULK3(A)* and *ULK3(B)*, amplifying different regions of the mRNA were used for *ULK3*. n(HKC strains)=9, n(SCC cell lines)=7, mean ± SEM, $P<0.01$, two-tailed unpaired t-test.

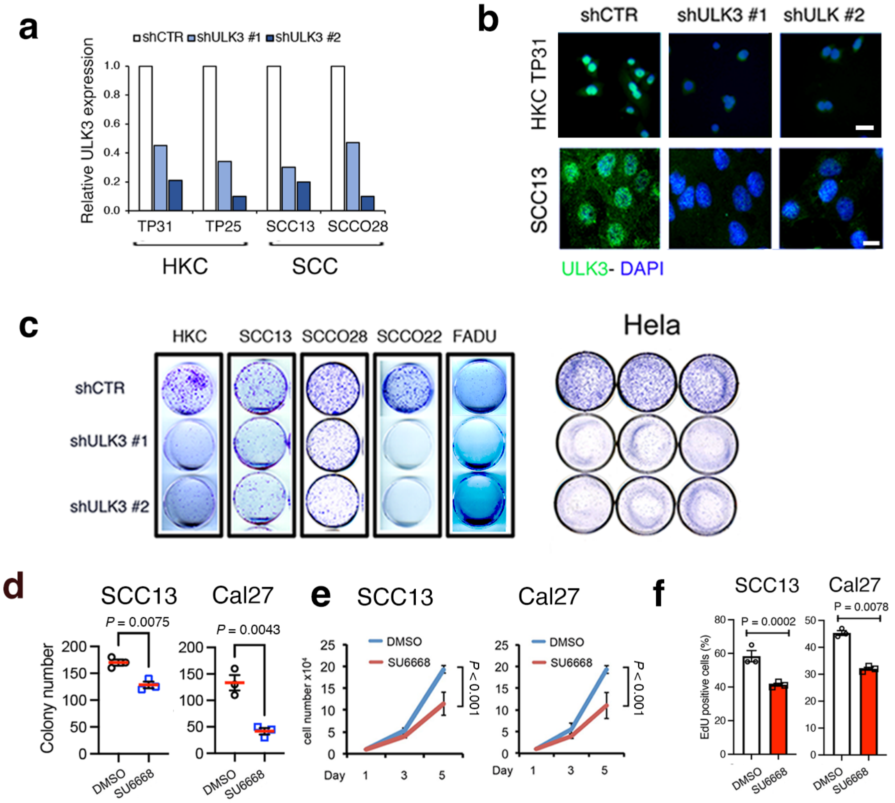

Supplementary Figure 2

a) Efficient downregulation of *ULK3* mRNA in HKCs and SCCs. RT-qPCR analysis of *ULK3* expression, normalized to *36ß4*, of two HKC strains (#TP31, #TP25) and two SCC cell lines infected with two *ULK3* silencing lentiviruses (shULK3 #1 and #2) or a control virus (shCTR) for one week.

b) Efficient downregulation of ULK3 protein in HKCs and SCCs. ULK3 immunofluorescence (green) in one HKC strain (TP31) infected with two *ULK3* silencing lentiviruses (shULK3 #1 and #2) or a control virus (shCTR) for one week. DAPI stained nuclei (blue). Scale bars 10 μM and 5μM

c) ULK3 silencing blocks HKC and SCC proliferation. Representative images of the colony assays dishes plus/minus ULK3 infection with two lentiviruses (shULK3 #1 and #2), or a control virus (shCTR), of the experiments shown in Fig. 2c.

d-f) Inhibition of SCCs cells growth by a ULK3 small-molecule inhibitor.

d) Colony formation assays of SCC13 and Cal27 cells treated with 10μM ULK3 inhibitor SU6668 [1] or DMSO vehicle for 7 days. Quantification of cell cultures, n(dishes)=3, mean ± SEM, P<0.001; two-tailed unpaired t-test.

e) SCC13 and Cal27 cells were detached and counted with a hemocytometer at days 3 and 5 from continuous treatment with 10μM ULK3 inhibitor SU6668 [1] or DMSO vehicle. n(dishes)=3, mean ± SEM, P<0.0001, two-tailed unpaired t-test.

f) DNA synthesis assays using 5-Ethynyl-2-deoxyuridine (EDU) of SCC13 and Cal27 cells treated with 10μM ULK3 inhibitor SU6668 [1] or DMSO vehicle for 48h. Quantification of cells pulse-labeled with EDU for 4h with DAPI to identify total cell number, n(dishes)=3, mean ± SEM, P<0.001, two-tailed unpaired t-test.

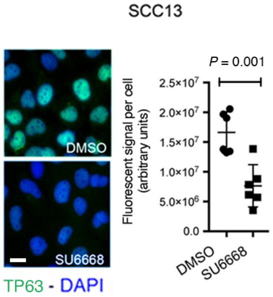

Supplementary Figure 3

a) Downmodulation of ULK3 activity with SU6668 inhibitor decreases TP63 levels. TP63 immunofluorescence (green) of SCC13 cells treated with ULK3 inhibitor SU6668 [1] for 72h, or DMSO vehicle as control. DAPI stained nuclei (blue). Representative image of the cell cultures and quantification of nuclear fluorescence signal intensity in individual cells, n(cells/condition)=60, mean ± SD, P<0.001, two-tailed unpaired t-test. Scale bar 5μM.

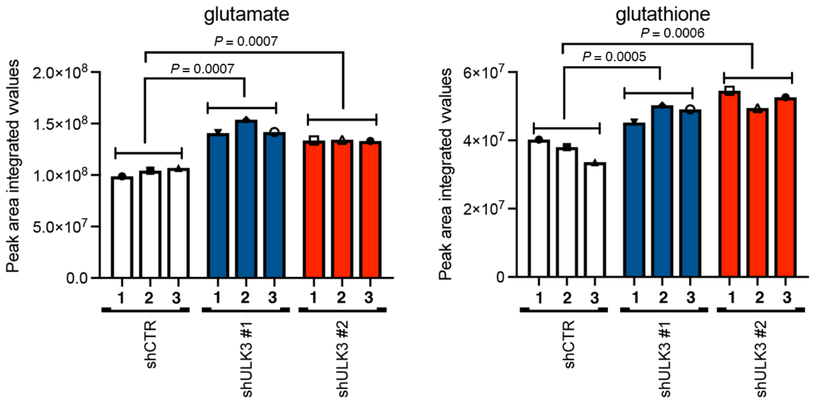

Supplementary Figure 4

a) Triplicate values of integrated peak area for glutathione and glutamate used for the metabolomics analysis in Fig 4g. SCC13 cells were infected in triplicate with two *ULK3* silencing lentiviruses, or a control virus. After the selection cells were expanded for a week before the extraction of metabolites. n(dishes)=3, glutathione $P<0.0005$ and glutamate $P<0.0007$, one way ANOVA.

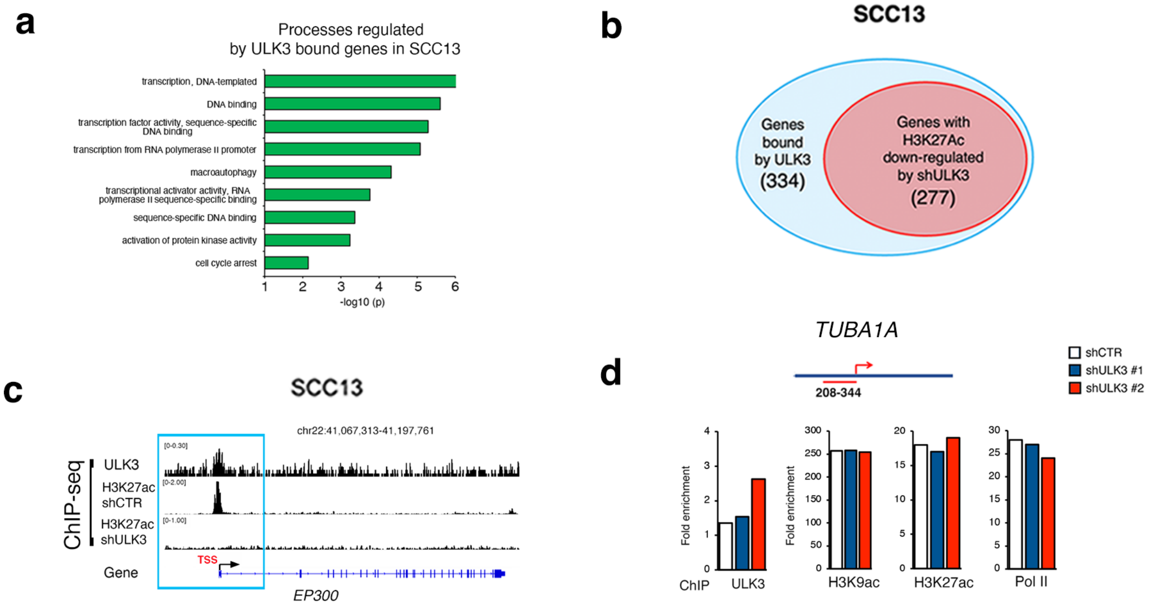

Supplementary Figure 5

a) Gene ontology analysis using Database for Annotation, Visualization and Integrated Discovery (David) software of processes regulated by the ULK3 bound genes as detected by ChIP seq analysis in SCC13 cells with anti-ULK3 antibodies, P<0.01, Fisher's exact test. The processes are ranked by significance (log10).

b) Euclidean diagram showing the number of ULK3-bound genes in SCC13 cells and the genes with reduced H3K27ac peaks in cells with *ULK3* silencing, as seen by ChIP-seq analysis with antibodies against ULK3 and H3K27ac, respectively.

c) Downregulation of the H3K27ac-binding peaks on EP300 gene, a transcriptional and epigenetic regulator bound by ULK3 in ChIP seq analysis. Graphic localization of the ULK3 and H3K27ac enriched peaks on EP300 gene, in SCC13 cells infected with control versus ULK3 silencing lentiviruses. Position of Transcriptional Start Sites (TSS) and exon (vertical blue lines) and intron transcribed regions were obtained from ENCODE and plotted using integrative genomic viewer software (IGV v 2.8.13). The blue square marks areas with loss of H3K27ac binding in cells with silenced ULK3.

d) *TUBA1A* gene is not bound by ULK3 in ChIP seq analysis or regulated by ULK3 silencing in SCC13 cells. (Findings relate as a negative control to those in the Fig. 5 experiments). Direct ChIP using the indicated antibodies, in SCC13 cells infected with control versus two ULK3 silencing lentiviruses, followed by qPCR amplification of the indicated chromatin area within *TUBA1A* promoter region (https://genome.ucsc.edu/). The promoter region of alpha-tubulin gene (*TUBA1A)* and the position of the amplified region (magenta bar) with the location of the transcriptional start site (TSS). Enrichment is calculated by normalizing to parallel ChIP with non-immune antibodies.

a

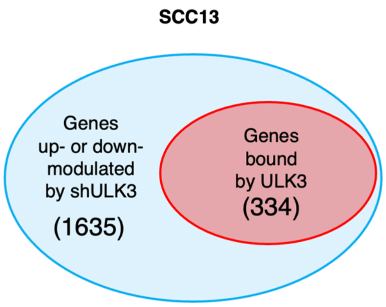

b

Genes similarly regulated
upon siPRTM1 and shULK3 in HKC

PRMT1vsULK3_UP	PRMT1vs ULK3_DOWN
CCDC80	ARSI
CPE	CDCA7
DDAH1	CDT1
DNAJB6	DTL
GBP1	ETS2
HGSNAT	FAR2
IFIT3	FKBP11
IL32	FOSL1
KLHL28	HPSE
NEFL	MCM7
RC3H2	MOCOS
RSAD2	PSMC3IP
SAMD9L	PTGS2
SAT1	SLC1A4
SPINK6	SYNJ2
VAV3	UHRF1

c

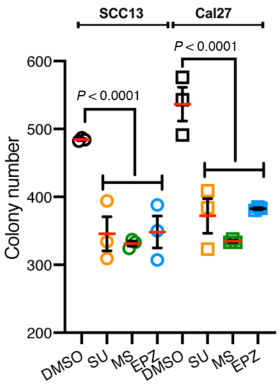

Supplementary Figure 6

a) Venn diagram of the genes up- or down- modulated upon ULK3 silencing in SCC13 cells, as determined by transcriptomic analysis (GSE: 183085), and of the genes bound by ULK3, determined by ChIP seq analysis with anti ULK3 antibodies in SCC13 cells (GSE: 183933).

b) List of genes similarly modulated by siRNA-mediated PRMT1 silencing in HKCs [2] and by shRNA-mediated silencing of *ULK3* in HKCs. The expression analysis of HKC with or without infection with two lentiviruses targeting *ULK3* expression, or a control virus, is described in Fig. 3a, b (GSE: 183084).

c) Effects of PRMT1 and PRMT5 versus ULK3 small-molecule inhibitors on SCC cell growth. Colony assays of two SCC cell lines plated at limited cell density and treated for one week with PRMT1 inhibitor MS-203 [3], PRMT5 inhibitor EPZ015666 [4], ULK3 inhibitor SU6668 [1] (positive control) or DMSO (vehicle, negative control). Colony number quantification, n(dishes)=3, mean ± SEM, $P<0.001$, one-way ANOVA.

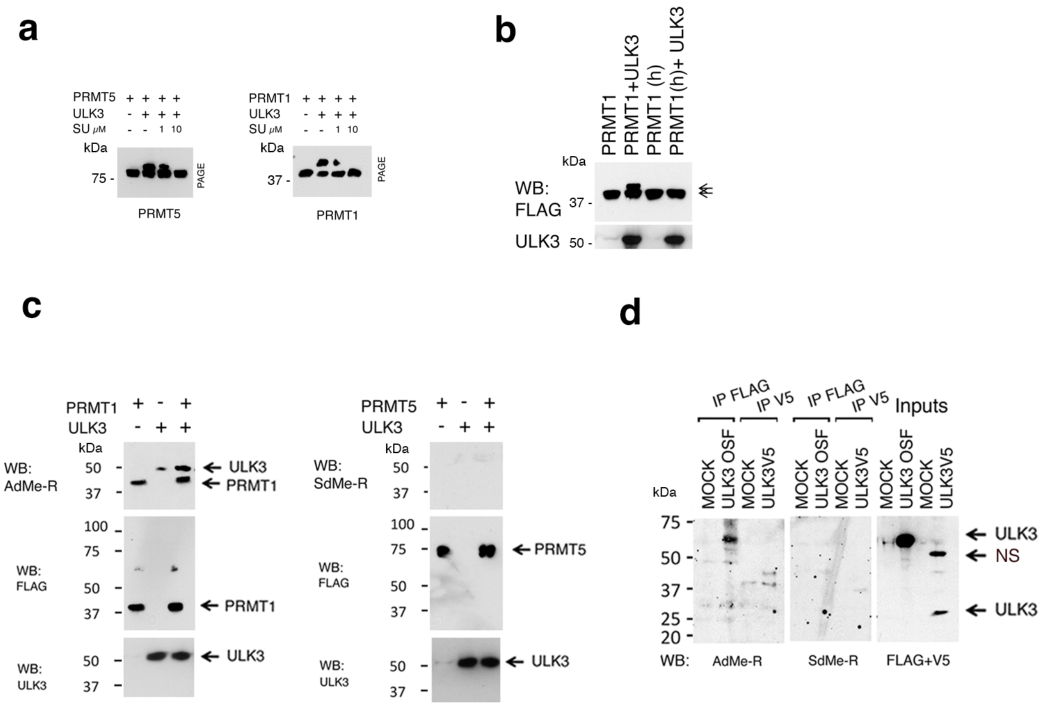

Supplementary Figure 7

a) ULK3 kinase assay using GFP as a substrate. GFP immunoblotting of ULK3 kinase reactions with recombinant HIS-tagged GFP protein used as a negative control. The kinase reactions were separated on a Phos-TAG gel before blotting. To detect ULK3 protein in the reaction, the same membrane was sequentially incubated with anti-ULK3 antibodies.

b) FLAG immunoblotting of ULK3 kinase reactions with PRMT1 plus/minus heating for 20 min at 90 C (depicted as h) to denature the proteins before the kinase assay, and separated on Phos-TAG gels. The membranes were sequentially incubated with anti-ULK3.

c) Anti asymmetric- or symmetric- dimethylated arginine motifs immunoblotting (AdMe-R and SdMe-R respectively) of in vitro methylation reactions incubating recombinant ULK3 with recombinant PRMT1 (left) or PRMT5 (right). The same amount of recombinant proteins were incubated singularly as controls, and the membranes were sequentially stained with anti-FLAG and ULK3 antibodies.

d) AdMe-R or SdMe-R immunoblotting of immunoprecipitations with anti-FLAG (OSF) or -V5 antibodies from HEK 293 cells transfected with either FLAG-tagged ULK3 (OSF)[5] or V5-tagged ΔCtULK3[6], versus mock-transfected cells. Inputs were stained simultaneously with anti FLAG and V5 antibodies (FLAG+V5).

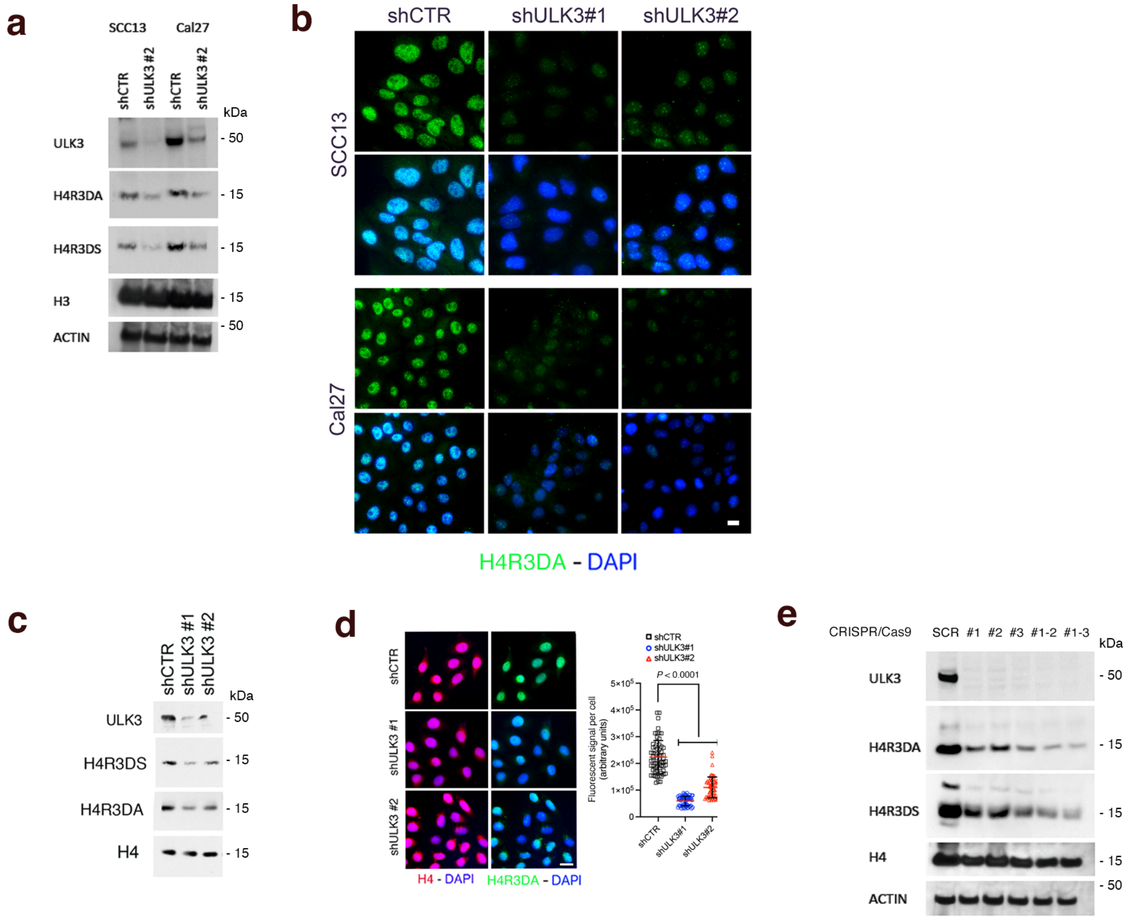

Supplementary Figure 8

a) *ULK3* silencing downmodulates arginine 3 dimethylation of histone 4 (H4R3). Asymmetrically (H4R3DA) or symmetrically (H4R3DS) dimethylated H4R3 immunoblotting in SCC13 and Cal27 cells with *ULK3* silencing for two weeks (shULK3), versus a control (shCTR). Sequential incubation with anti total histone -H3 (H3) and -ß-ACTIN (ACTIN) antibodies.

b) *ULK3* silencing downmodulates H4R3 dimethylation. Anti-H4R3DA antibodies (green) immunofluorescence in SCC13 and Cal27 cells plus/minus *ULK3* silencing with two lentiviruses (shULK3 #1 and #2), or a control virus (shCTR), for two weeks. DAPI stained the nuclei (blue). Scale bar 5μM.

c) H4R3DA, H4R3DS, total H4 and ULK3 immunofluorescence in HeLa cells plus/minus *ULK3* silencing with two lentiviruses, or a control virus for one week.

d) Representative immunofluorescence images of HeLa cells plus/minus *ULK3* silencing as in the previous panel with anti-H4R3DA (green) and H4 (magenta) antibodies, with DAPI nuclear staining (blue), and quantification of individual cells nuclear fluorescence signal intensity, mean ± SD, n(cells)>60, P<0.0001, one-way ANOVA. Scale bar 5μM**.**

e) *ULK3* gene deletion in HEK293 cells results in loss of H4R3 dimethylation. ULK3, H4R3DA, H4R3DS, total H4 and ß-ACTIN immunoblotting of HEK293T cells one week after the infection with a lentivirus encoding for three separate RNA guides (gRNA) targeting ULK3 gene, #1/#2/#3 alone, or #1+#2 and #1+#3 in combination, or with a scrambled control gRNA (SCR). Cas9 enzyme was co-expressed in the same vector.

TABLE S1- shRNA sequences (Sigma-Mission) used for RNA-interference experiments

target	# id	sequence
ULK3 #1	37419	CCGGCGTGTCTTCATGCAGCAATTACTCGAGTAATTGCTGCATGAAGACACGTT TTTG
ULK3 #2	37420	CCGGGCAGACTTTGGTTTCGCACAACTCGAGTTGTGCGAAACCAAAGTCTGCTT TTTG
ULK3 #3	37422	CCGGTCCTGTATGGTGAGACCTCTTCTCGAGAAGAGGTCTCACCATACAGGATT TTTG

TABLE S2 - Sequences of the oligonucleotides for qPCR experiments

Gene		
ULK1	TCGAGTTCTCCCGCAAGG	CGTCTGAGACTTGGCGAGGT
ULK2	TGGGTCCTCCCAACTATCTACAAGT	CGAGATGTTGTGTGGCACCAA
ULK3	TGAAGGAGCAGGTCAAGATGAGG	GCTACGAACAGATTCCGACAGTCC
ULK4	ACTAACCCCAGGGCCTAGAA	ACCACAGGCAGTGCTAACCT
p21	GATTAGCAGCGGAACAAGGA	CAACTACTCCCAGCCCCATA
ß-Actin	GTTGTCGACGACGAGCG	GCACAGAGCCTCGCCTT
36ß4	GCAATGTTGCCAGTGTCTGT	GCCTTGACCTTTTCAGCAAG
GLI1	GTGCAAGTCAAGCCAGAACA	GGGGGTAATGGGAAAAGAGA
GLI2	CAGTGTGCATGCCTGGTATC	TGGAATTTGGAACTGGCTTC
PRMT1	GAGAATTTTGTAGCCACCTTGG	CCTGGCCACAGGACACTT
PRMT5	TGAACACAGTGCTTCATGGCTTCG	GAATTGCTGCATCGCCAGAAACGC
MYC	CCTGGTGCTCCATGAGGAGAC	CAGACTCTGACCTTTTGCCAGG
KRT10	GAAAAGCATGGGCAACTCACA	TGTCGATCTGAAGCAGGATG
KRT14	CCAGTTCTCCTCTGGATCGCAG	GGATCTTCCAGTGGGATCTGTGTCCA
FLG	GCTGAAGGAACTTCTGGAAAAGG	GTTGTGGTCTATATCCAAGTGATC
IVL	TGCCTGAGCAAGAATGTGAG	TGCTCTGGGTTTTCTGCTTT
FOXM1	CACCCCAGTGCCAACCGCTACTTG	AAAGAGGAGCTATCCCCTCCTCAG
LDHA	CAGCTTGGAGTTTGCAGTTAC	TGATGGATCTCCAACATGG
PKM	CTATCCTCTGGAGGCTGTGC	CCATGAGGTCTGTGGAGTGA
TIGAR	CTGACTGAAACTCGCTAAGG	CAGAACTAGCAGAGGAGAGA
SLC2A1	CTTTGTGGCCTTCTTTGAAGT	CCACACAGTTGCTCCACAT
**ChIP**		
LDHA a	CGGCCGTTTCCTCATTTCTT	ATTTGGTGGCGGTGACTCTA
LDHA b	TCAGCAAGAATACAGGCCCA	TCCTGGCCTCAAGTGATCTG
LDHA c	CAGTGAGTAGGAGGCAGAGG	CTGCTAGCTTCACTGAACGG
LDHA d	GCATAGCTCCAGATTGCCTC	GAAAGGTGGGCGGAAATCAG
PKM a	AGTCATCTTCCCCACAGAGC	AGGAGATGGAGGTCAGGACT
PKM b	CCTGGGTCTGGAGTACGTAG	TAATGCTGGGAGTGGGGTAC
PKM c	GAGGAAGAGGATGGGACCAG	TGCTCTTCAGAAGTCCCCAG
PKM d	GGCAGTAGGGAGAACTAGGG	CATTCGCTCTGCAGGATTCC
FOXM1 a	TTCCTGACCAGCCCGTTATT	GGCCCTTTTCCTGGTTCTTG
FOXM1 b	CTCCTCTGCTCCCCTTTTCA	GCTGAGGTAGGGTTCATGGT
TUB1A1	GGGGACTGGGGTTAGAAGAC	GGCTGGCAGTCATTTTCCTT
**Surveyor**		
OLIGO_1	CGTTGTTAACTTCTAGGCTC	CCTTGTCTGACTGGAAAGGC
OLIGO_2	ATAAAGTGTGTAGCCAAGAA	TCTAATGTTTTCCATATAAG
OLIGO_3	GAAAAGGTGCCCAAGGAGCT	AAGGGCCTGCAAAATGGGCA
CTR	AAGTTGTTCTGATGGGGCAG	CCTGCCCTCCCCTCCAGTAT

TABLE S3- Sequences of the siRNA and LNAs

**Oligonucleotides**	
siCTR	Ambion # 4390846
siULK3 #1	Ambion # 4992420
siULK3 #2	Ambion # 4992420
**Locked antisense nucleotides**	
LNA SCR	IDT ASO NC5
LNA #1	IDT ASO 155_2
LNA #2	IDT ASO 155_6
LNA #3	IDT ASO 245_4

TABLE S4- Vectors and recombinant proteins

**Recombinant DNA**	
ULK3 wt, deletion/mutation and control vectors:	
ULK3 wt OSF	BioMMED LSU
ULK3 K139R OSF	BioMMED LSU
ULK3 K44H OSF	BioMMED LSU
pLenti6 V5 ULK3	ThermoLife # K495510
pLenti GFP ULK3	Addgene # 17445
pLenti HA ULK3	ABMGOOD
ULK3 CrispR and control vectors:	
pLenti_U6_sgRNA_SFFV_Cas9_2A_Puro #Scrambled	ABMGOOD
pLenti_U6_sgRNA_SFFV_Cas9_2A_Puro #1	ABMGOOD # K2587605
pLenti_U6_sgRNA_SFFV_Cas9_2A_Puro #2	ABMGOOD # K2587605
pLenti_U6_sgRNA_SFFV_Cas9_2A_Puro #3	ABMGOOD # K2587605
pLENTI EGFP	Tiscornia et al.

TABLE S5 - Antibodies used

REAGENT or RESOURCE	SOURCE	IDENTIFIER	DILUTIONs
**Antibodies**			
ß-actin, rabbit	Cell Signaling # 13E5	AB_10694076	WB:1:1000
γ-tubulin, rabbit	Sigma # GTU-88	AB_523854	WB:1:1000
TP63, mouse	Santa Cruz # 8343	AB_653763	WB:1:1000; IF 1:100
Ki67, rabbit	Abcam # 15580	AB_443209	IF 1:100
Pan KRT, mouse	Abcam # 7753	AB_306047	IF 1:100
H4R3DS, rabbit,	EpiGentek# 10019-4R3DS	N/A	WB:1:1000; IF 1:100
H4R3DA , abbit,	EpiGentek# 10019-4R3DA	N/A	WB:1:1000; IF 1:100
H4, rabbit	Millipore # 07-108	AB_11210265	WB:1:1000; IF 1:100
H3, rabbit	Cell Signaling # 4620	AB_1904005	WB:1:1000; ChIP 1:100
H3K27ac, rabbit	Abcam # 4729	AB_2118291	ChIP 1:100
KRT10, rabbit,	Covance # 19054	N/A	WB:1:1000; IF 1:100
TP63, rabbit	Cell Signaling # 13109	AB_2637091	WB:1:1000; IF 1:100
PRMT1, mouse	Santa Cruz # 166963	AB_10610884	WB:1:1000; IF 1:100; PLA 1:100
PRMT5, mouse	Santa Cruz # 424245	N/A	WB:1:1000; IF 1:100; PLA 1:100
PRMT1, rabbit	Cell Signaling # 2449	AB_2237696	WB:1:1000; IP 1:100; ChIP 1:100
PRMT5, rabbit	Cell Signaling # 79998	AB_2799945	WB:1:1000; IP 1:100; ChIP 1:100
ULK3, rabbit	Santa Cruz # 137897	AB_11150315	WB:1:1000; IF 1:100; IP 1:100
ULK3, mouse	Santa Cruz # 517373	N/A	WB:1:1000; IF 1:100; IP 1:100
ULK3, rabbit	Abcam # EPR4888	AB_10972508	WB:1:1000
ULK3, mouse	MyBios. # MBS9200567	AB_2754977	WB:1:1000; IF 1:100; PLA 1:50
ULK3, rabbit	Atlas Abs # HPA040474	AB_2677003	IF 1:100
VIMENTIN, mouse	Abcam # 20346	AB_445527	IF 1:100
p21, mouse	Cell Signaling # 2947	AB_823586	WB:1:1000
p63, rabbit	Abcam # 735	AB_305870	WB:1:1000
GLS1, rabbit	Cell Signaling # 88964	AB_2800133	WB:1:1000
PKM1, rabbit	Cell Signaling # 7067	AB_2715534	WB:1:1000
Phospho Ser/Thr, rabbit	Abcam #17464	AB_443891	PLA 1:100
H3K9ac, rabbit	Upstate #06-942	AB_310308	ChIP 1:100
Pol II, rabbit	Upstate #05-623	AB_309852	ChIP 1:100
LAMIN B1, mouse	Santa Cruz #374015	AB_10947408	WB 1:1000
FLAG, mouse	Sigma #F104	AB_438695	WB 1:1000; IP 1:100
HIS TAG, mouse	Cell Signaling #2365	N/A	WB 1:1000
Symmetric DMe-ARG, rabbit	Cell Signaling #13222	AB_2714013	WB 1:1000
Asymmetric DMe-ARG, rabbit	Cell Signaling #13522	AB_2665370	WB 1:1000
Involucrin, rabbit	Abcam #227530	N/A	WB 1:1000
Non immune IgG, mouse	Abcam # 5415	N/A	WB:1:1000; IF 1:100; PLA 1:100; IP 1:100
Non immune IgG, rabbit	Abcam # 2729	N/A	WB:1:1000; IF 1:100; PLA 1:100; IP 1:100

Supplementary references

1. Kasak, L. *et al.* Characterization of Protein Kinase ULK3 Regulation by Phosphorylation and Inhibition by Small Molecule SU6668. *Biochemistry* **57**, 5456-5465, doi:10.1021/acs.biochem.8b00356 (2018).
2. Bao, X. *et al.* CSNK1a1 Regulates PRMT1 to Maintain the Progenitor State in Self-Renewing Somatic Tissue. *Dev Cell* **43**, 227-239.e225, doi:10.1016/j.devcel.2017.08.021 (2017).
3. Eram, M. S. *et al.* A Potent, Selective, and Cell-Active Inhibitor of Human Type I Protein Arginine Methyltransferases. *ACS Chem Biol* **11**, 772-781, doi:10.1021/acschembio.5b00839 (2016).
4. Chan-Penebre, E. *et al.* A selective inhibitor of PRMT5 with in vivo and in vitro potency in MCL models. *Nature chemical biology* **11**, 432-437, doi:10.1038/nchembio.1810 (2015).
5. Caballe, A. *et al.* ULK3 regulates cytokinetic abscission by phosphorylating ESCRT-III proteins. *Elife* **4**, e06547, doi:10.7554/eLife.06547 (2015).
6. Goruppi, S. *et al.* The ULK3 Kinase Is Critical for Convergent Control of Cancer-Associated Fibroblast Activation by CSL and GLI. *Cell Rep* **20**, 2468-2479, doi:10.1016/j.celrep.2017.08.048 (2017).
7. Tiscornia, G., Singer, O., Ikawa, M. & Verma, I. M. A general method for gene knockdown in mice by using lentiviral vectors expressing small interfering RNA. *Proc Natl Acad Sci U S A* **100**, 1844-1848, doi:10.1073/pnas.0437912100 (2003).
