# Supplementary material for: The ULK3 kinase is a determinant of keratinocyte self-renewal and tumorigenesis targeting the arginine methylome
Source: Nat Commun. 2023 Feb 16;14:887. doi: 10.1038/s41467-023-36410-6 (PMC9935893; doi:10.1038/s41467-023-36410-6)
Supplement: Supplementary file 3 — Reporting Summary [file 41467_2023_36410_MOESM3_ESM.pdf]

nature portfolio

Corresponding author(s): Gian Paolo Dotto and Sandro Goruppi

Last updated by author(s): December 10, 2022

Reporting Summary

Nature Portfolio wishes to improve the reproducibility of the work that we publish. This form provides structure for consistency and transparency in reporting. For further information on Nature Portfolio policies, see our Editorial Policies and the Editorial Policy Checklist.

Statistics

For all statistical analyses, confirm that the following items are present in the figure legend, table legend, main text, or Methods section.

n/a	Confirmed	
☐	☒	The exact sample size (*n*) for each experimental group/condition, given as a discrete number and unit of measurement
☐	☒	A statement on whether measurements were taken from distinct samples or whether the same sample was measured repeatedly
☐	☒	The statistical test(s) used AND whether they are one- or two-sided<br>*Only common tests should be described solely by name; describe more complex techniques in the Methods section.*
☐	☒	A description of all covariates tested
☐	☒	A description of any assumptions or corrections, such as tests of normality and adjustment for multiple comparisons
☐	☒	A full description of the statistical parameters including central tendency (e.g. means) or other basic estimates (e.g. regression coefficient) AND variation (e.g. standard deviation) or associated estimates of uncertainty (e.g. confidence intervals)
☐	☒	For null hypothesis testing, the test statistic (e.g. *F*, *t*, *r*) with confidence intervals, effect sizes, degrees of freedom and *P* value noted<br>*Give P values as exact values whenever suitable.*
☒	☐	For Bayesian analysis, information on the choice of priors and Markov chain Monte Carlo settings
☒	☐	For hierarchical and complex designs, identification of the appropriate level for tests and full reporting of outcomes
☒	☐	Estimates of effect sizes (e.g. Cohen's *d*, Pearson's *r*), indicating how they were calculated

Our web collection on statistics for biologists contains articles on many of the points above.

Software and code

Policy information about availability of computer code

Data collection	Nikon NIS elements, NDP2 viewer and Zeiss Zen softwares for microscopy.
Data analysis	ImageJ2 v2.3.0/1.53q, Prism 9, Microsoft Excel 365, Integrative Genomic Viewer 2.8.13 (MIT/Broad), Morpheus heat map plotter (MIT), Gene expression profile interactive analysis GEPIA, DAVID gene ontology software, Galaxy web tools for biomedical data research, MACS2, PeaksAnnotator, Bowtie2 Version 2.3.0, gene set enrichment analysis GSEA 4.0.1, Metabolomics data analysis Metaboanalyst

For manuscripts utilizing custom algorithms or software that are central to the research but not yet described in published literature, software must be made available to editors and reviewers. We strongly encourage code deposition in a community repository (e.g. GitHub). See the Nature Portfolio guidelines for submitting code & software for further information.

Data

Policy information about availability of data

All manuscripts must include a data availability statement. This statement should provide the following information, where applicable:

- Accession codes, unique identifiers, or web links for publicly available datasets
- A description of any restrictions on data availability
- For clinical datasets or third party data, please ensure that the statement adheres to our policy

The transcriptomic (HKCs and SCC13 cells) and ChIP-seq data generated in this study have been deposited in GEO under the accession codes GSE183084 (https://www.ncbi.nlm.nih.gov/geo/query/acc.cgi?acc=GSE183084), GSE183085 (https://www.ncbi.nlm.nih.gov/geo/query/acc.cgi?acc=GSE183085) and GSE183933 (https://www.ncbi.nlm.nih.gov/geo/query/acc.cgi?acc=GSE183933), respectively. The metabolomics data have been deposited in MetaboLights as MTBLS5155 (https://www.ebi.ac.uk/metabolights/MTBLS5155/descriptors). For the analysis we used Ch38 human genome assembly (https://www.ncbi.nlm.nih.gov/assembly/GCF_000001405.26/).

Sequences for shRNA, siRNA, LNAs, CRIPR/cas9, PCR oligos and antibodies used are in Supplementary TABLEs 1-5. Source data underlying Figures 1a-h, 2a-f, 3a-j, 4b-g, 5c-f, 6b-g, 7a-g, 8a, b, d-h, 9a-g, 10a-i and Supplementary Figures S1d, S2a, S2d, S2e, S2f,S4, S5d, S6e, S7a-c, S8s, S8d are provided with this paper.
There is no restriction on data availability.

Field-specific reporting

Please select the one below that is the best fit for your research. If you are not sure, read the appropriate sections before making your selection.

☒ Life sciences ☐ Behavioural & social sciences ☐ Ecological, evolutionary & environmental sciences

For a reference copy of the document with all sections, see nature.com/documents/nr-reporting-summary-flat.pdf

Life sciences study design

All studies must disclose on these points even when the disclosure is negative.

Sample size	Sample size was determined using power calculation allowing the use of statistical analysis.<br>Statistical significance of differences between experimental groups and controls was assessed by two-tailed unpaired or paired t-test, and one way ANOVA, as indicated in the legends. In all cases, p values < 0.05 were considered as statistically significant.
Data exclusions	No data or sample were excluded from the analysis.
Replication	The experiments were replicated in three or more patient-derived SCC cell lines or in independently derived primary keratinocyte strains. Three AKs and six Skin SCC patient-derived samples and normal unaffected areas were used. All attempts were successful.
Randomization	No randomization was adopted
Blinding	Considering the multiple aspects of the study, complete blinding of the investigators was not possible for the data collection, nor relevant considering the cross-check of the results. Infact, the experimental evidence and data analysis were independently replicated by several Investigators involved in the study (in the two laboratories of the PI) by using different lentivirus preparations, cell line stocks and HKC strains. The in vivo experiments were carried out as a team by four investigators.

Reporting for specific materials, systems and methods

We require information from authors about some types of materials, experimental systems and methods used in many studies. Here, indicate whether each material, system or method listed is relevant to your study. If you are not sure if a list item applies to your research, read the appropriate section before selecting a response.

Materials & experimental systems

n/a	Involved in the study	
☐	☒	Antibodies
☐	☒	Eukaryotic cell lines
☒	☐	Palaeontology and archaeology
☐	☒	Animals and other organisms
☐	☒	Human research participants
☐	☒	Clinical data
☒	☐	Dual use research of concern

Methods

n/a	Involved in the study	
☐	☒	ChIP-seq
☒	☐	Flow cytometry
☒	☐	MRI-based neuroimaging

Antibodies

Antibodies used

ANTIBODY, SPECIES, SOURCE, CATALOG #, IDENTIFIER, USE and DILUTION
"ß-actin", rabbit, Cell Signaling # 13E5 , AB_10694076, WB:1:1000
"gamma-tubulin", rabbit, Sigma # GTU-88, AB_523854, WB:1:1000
"TP63", mouse, Santa Cruz # 8343, AB_653763, WB:1:1000; IF 1:100
"Ki67", rabbit, Abcam # 15580, AB_443209, IF 1:100
"Pan KRT", mouse, Abcam # 7753, AB_306047, IF 1:100
"H4R3DS", rabbit, EpiGentek# 10019-4R3DS, N/A, WB:1:1000; IF 1:100
"H4R3DA", rabbit, EpiGentek# 10019-4R3DA, N/A, WB:1:1000; IF 1:100
"H4", rabbit, Millipore # 07-108, AB_11210265, WB:1:1000; IF 1:100
"H3', rabbit, Cell Signaling # 4620, AB_1904005, WB:1:1000; ChIP 1:100
"H3K27ac", rabbit, Abcam # 4729, AB_2118291, ChIP 1:100
"KRT10", rabbit, Covance # 19054, N/A, WB:1:1000; IF 1:100
"TP63", rabbit, Cell Signaling # 13109, AB_2637091, WB:1:1000; IF 1:100
"PRMT1", mouse, Santa Cruz # 166963, AB_10610884, WB:1:1000; IF 1:100; PLA 1:100

"PRMT5", mouse, Santa Cruz # 424245, N/A, WB:1:1000; IF 1:100; PLA 1:100
"PRMT1", rabbit, Cell Signaling # 2449, AB_2237696, WB:1:1000; IP 1:100; ChIP 1:100
"PRMT5", rabbit, Cell Signaling # 79998, AB_2799945, WB:1:1000; IP 1:100; ChIP 1:100
"ULK3", rabbit, Santa Cruz # 137897, AB_11150315, WB:1:1000; IF 1:100; IP 1:100
"ULK3", mouse, Santa Cruz # 517373, N/A, WB:1:1000; IF 1:100; IP 1:100
"ULK3", rabbit, Abcam # EPR4888, AB_10972508, WB:1:1000
"ULK3", mouse, MyBios. # MBS9200567, AB_2754977, WB:1:1000; IF 1:100; PLA 1:50
"ULK3", rabbit, Atlas Abs # HPA040474, AB_2677003, IF 1:100
"VIMENTIN", mouse, Abcam # 20346 , AB_445527, IF 1:100
"p21", mouse, Cell Signaling # 2947, AB_823586, WB:1:1000
"p63", rabbit, Abcam # 735, AB_305870, WB:1:1000
"GLS1", rabbit, Cell Signaling # 88964, AB_2800133, WB:1:1000
"PKM1", rabbit, Cell Signaling # 7067, AB_2715534, WB:1:1000
"Phospho Ser/Thr", rabbit, Abcam #17464, AB_443891, PLA 1:100
"H3K9ac", rabbit, Upstate #06-942, AB_310308, ChIP 1:100
"Pol II", rabbit, Upstate #05-623, AB_309852, ChIP 1:100
"LAMIN B1", mouse, Santa Cruz #374015, AB_10947408, WB 1:1000
"FLAG", mouse, Sigma #F104, AB_438695, WB 1:1000; IP 1:100
"HIS TAG", mouse, Cell Signaling #2365, N/A, WB 1:1000
"Symmetric DMe-ARG", rabbit, Cell Signaling #13222, AB_2714013, WB 1:1000
"Asymmetric DMe-ARG", rabbit, Cell Signaling #13522, AB_2665370, WB 1:1000
"Involucrin", rabbit, Abcam #227530, N/A, WB 1:1000
"Non immune IgG", mouse, Abcam # 5415, N/A, WB:1:1000; IF 1:100; PLA 1:100; IP 1:100
"Non immune IgG", rabbit, Abcam # 2729, N/A, WB:1:1000; IF 1:100; PLA 1:100; IP 1:100

Validation

All antibodies underwent an application-specific validation by the companies. We indicate the Research Resource Identifier (https://scicrunch.org/resources), providing a link to the validation and previous published use of each antibody. All ULK3 antibodies were confirmed by gene silencing (siRNA and shRNA) and CrispR/Cas9 mediated gene deletion, coupled to human ULK3 cDNA-expression vector. Mouse anti PRMT1 and PRMT5 antibodies (# 166963, # 424245) were validated by siRNA silencing andrabbit anti PRMT1 and PRMT5 antibodies (# 2449, # 79998) validated by using human cDNA-expression vector. Three antibodies (# 137897, # 517373,# EPR4888) were used for ULK3 and two for PRMT1/PRMT5 (# 166963, # 424245, # 2449, # 79998) to confirm the detected signal/localization in WB and IF. Anti ULK3 used for tissue arrays (https://www.proteinatlas.org/ENSG00000140474-ULK3/summary/antibody) was validated by IHC, WB and peptide array. All other antibodies (used in ChIP, WB and IF) were previously tested and published by the authors or other research groups.

Eukaryotic cell lines

Policy information about cell lines

Cell line source(s)

Primary HKCs were prepared from discarded human samples from abdominoplasty at the CBRC, Massachussetts General Hospital, Boston with Institutional approval (IRB #2018P003156). All HKC strains were identified by a progressive number and two letters
identifying the operators.
primary HKC strains from discarded skin: strains GB #1- GB#14
primary HKC strains from discarded skin strains TP#24, TP#25, TP#31
SCC12 RRID:CVCL_4026
SCC13 RRID:CVCL_4029
Cal27 RRID:CVCL_1107
Cal33 RRID:CVCL_1108
Fadu RRID:CVCL_1218
SCCO13 RRID:CVCL_C06
SCCO22 RRID:CVCL_7731
SCCO28 RRID:CVCL_7738
HeLa ATCC RRID:CVCL_0030
293T ATCC RRID:CVCL_0063

Authentication

Primary HKC were derived from discarded healthy donor skin and were tested by IF to be vimentin negative and Keratin-positive.
All SCC cell lines were commercially available and we indicated the Research Resource identifier linking to previous publications. Independent freeze-down vials and independent vials in the two laboratories of the PI were used for SCC13, Cal27, SCCO28, SCCO22 and FaDu cells. Morphology, presence of IF markers (keratins, positive), the presence of keratinocyte-specific gene by PCR with human-specific primers were used for validation. New ATTC purchased vials was used for Hela cells.

Mycoplasma contamination

All cell lines and keratinocyte strains were routinely checked for the absence of mycoplasma by HOECHST staining without permeabilization of parallel cultures. Observation at high magnification (630x) showed that all were negative.

Commonly misidentified lines
(See ICLAC register)

none

Animals and other organisms

Policy information about studies involving animals; ARRIVE guidelines recommended for reporting animal research

Laboratory animals	10 week-old female immunodeficient mice NOD/SCID IL2Rg chain null Jackson Laboratories<br>8 to 10-week-old female immunodeficient mice NOD/SCID (CB17sc-m) mice Taconic
Wild animals	none
Field-collected samples	none
Ethics oversight	All the animal procedures were performed as approved by the MGH IACUC protocol 2004N000170.

Note that full information on the approval of the study protocol must also be provided in the manuscript.

Human research participants

Policy information about studies involving human research participants

Population characteristics	*Describe the covariate-relevant population characteristics of the human research participants (e.g. age, gender, genotypic information, past and current diagnosis and treatment categories). If you filled out the behavioural & social sciences study design questions and have nothing to add here, write "See above."*
Recruitment	*Describe how participants were recruited. Outline any potential self-selection bias or other biases that may be present and how these are likely to impact results.*
Ethics oversight	*Identify the organization(s) that approved the study protocol.*

Note that full information on the approval of the study protocol must also be provided in the manuscript.

Clinical data

Policy information about clinical studies

All manuscripts should comply with the ICMJEguidelines for publication of clinical research and a completedCONSORT checklist must be included with all submissions.

Clinical trial registration	not applicable
Study protocol	Institutional Review Board (IRB# 2018P003156), Massachusetts General Hospital, Boston
Data collection	Human skin samples of actinic keratosis (AK), squamous cell carcinoma (SCC) and normal matched controls of nearby non-affected areas were obtained at the Department of Dermatology, Massachusetts General Hospital, as discarded parts not needed for diagnosis. All samples were given a progressive number and processed as approved by the IRB .<br>Both male and female skin for HKCs and SCCs were used, as available.<br><br>• The head and neck, lung and cervical data in Fig. 1a are based upon data generated by the TCGA Research Network: www.cancer.gov/tcga.<br><br>• Commercially available tissue arrays with normal or adjacent matched tissues as controls of skin squamous cell carcinoma (SK483), head and neck tumors (HN801c) and cervical squamous carcinomas (CR484) were purchased from US Biomax.
Outcomes	*Describe how you pre-defined primary and secondary outcome measures and how you assessed these measures.*

ChIP-seq

Data deposition

- [x] Confirm that both raw and final processed data have been deposited in a public database such as GEO.
- [x] Confirm that you have deposited or provided access to graph files (e.g. BED files) for the called peaks.

Data access links<br>*May remain private before publication.*	https://www.ncbi.nlm.nih.gov/geo/query/acc.cgi?acc=GSE183933
Files in database submission	GSM5574522 SCC13 cells input DNA<br>GSM5574523 SCC13 cells ChIP ULK3Ab<br>GSM5574524 SCC13 shCTR cells input<br>GSM5574525 SCC13 shULK3 cells input<br>GSM5574526 SCC13 shCTR ChIP H3K27ac<br>GSM5574527 SCC13 shULK3 ChIP H3K27ac

Genome browser session
(e.g. UCSC)

https://tinyurl.com/y3fadfom (IGV)

Methodology

Replicates

Chromatin prepared from SCC13 cells by using a simple ChIP assay kit (Cell Signaling), was immunoprecipitated using 10 µg anti ULK3 or non immune control antibodies (both MAbs), followed by quantification of the DNA by fluorometry on a Qubit system (Invitrogen) and quality/size determination using the Bioanalyzer assay (Agilent). Chromatin from SCC13 plus/minus ULK3 silencing was similarly immunoprecipitated using 5µg of anti H3K27ac antibodies (Abcam), or non-immune control antibodies (Abcam), and analyzed as above.

Sequencing depth

Sample Reads Clean_reads Mapped Unique_mapped frag_size
Input pair 20440987 20086784(98.27%) 18886875(94.03%) 202 Calculated_from_BAM
Input read1 20440987 20440572(100.00%) 18980778(92.86%) 202 Calculated_from_BAM
Input read2 20440987 20440566(100.00%) 18950321(92.71%) 202 Calculated_from_BAM
IP_Ulk3 pair 19742279 19256317(97.54%) 18137183(94.19%) 192 Calculated_from_BAM
IP_Ulk3 read1 19742279 19708808(99.83%) 18222792(92.46%) 192 Calculated_from_BAM
IP_Ulk3 read2 19742279 19708519(99.83%) 18188765(92.29%) 192 Calculated_from_BAM
Input_Ctl pair 24273535 23834354(98.19%) 22448436(94.19%) 3501996(15.60%) 203 Calculated_from_BAM
Input_Ctl read1 24273535 24273182(100.00%) 22579201(93.02%) 3511903(15.55%) 203 Calculated_from_BAM
Input_Ctl read2 24273535 24273120(100.00%) 22541254(92.87%) 203 Calculated_from_BAM
IP3A_shCtl pair 20668253 20413931(98.77%) 19794912(96.97%) 199 Calculated_from_BAM
IP3A_shCtl read1 20668253 20659911(99.96%) 19873684(96.19%) 199 Calculated_from_BAM
IP3A_shCtl read2 20668253 20659908(99.96%) 19837148(96.02%) 199 Calculated_from_BAM
Input_sh1 pair 20427140 19994859(97.88%) 18787067(93.96%) 3059555(16.29%) 197 Calculated_from_BAM
Input_sh1 read1 20427140 20425074(99.99%) 18881658(92.44%) 3067222(16.24%) 197 Calculated_from_BAM
Input_sh1 read2 20427140 20425273(99.99%) 18848730(92.28%) 197 Calculated_from_BAM
IP3A_shULK3 pair 22323440 21364126(95.70%) 19914249(93.21%) 204 Calculated_from_BAM
IP3A_shULk3 read1 22323440 22244207(99.65%) 20032989(90.06%) 204 Calculated_from_BAM
IP3A_shULK3 read2 22323440 22244290(99.65%) 19996991(89.90%) 204 Calculated_from_BAM

Single-end sequencing using MACS2 software to predict the frag_sizes of IP experiment.

Antibodies

Non immune IgG, mouse Abcam # 5415 N/A
ULK3, mouse Santa Cruz # 517373 N/A
H3K27ac, rabbit Abcam # 4729 AB_2118291

Peak calling parameters

Construction of libraries from ChIPs samples, QCs, processing and analysis of the data was performed by Novogene Co Ltd.
The initial image data produced by High-throughput sequencing Illumina platform was converted to sequenced reads by CASAVA Base Calling
The mapping of the reads to the reference genome hg38 was performed using BWA software
Duplicates were labeled using SAMBLAST and mapping quality value was calculated with MAPQ
The peak calling using was made using MACS2 software (and to calculate the number of peaks, the peak width and its distribution, and find the peak related genes)

Data quality

First step: check the raw data quality using FastQC.
The procedure for data trimming is in the following:
(1) Discard the reads with low quality (proportion of low quality bases larger than 50%);
(2) Discard the reads with N ratio (unsure base) larger than 15%;
(3) Discard the reads with adaptor at the 5'-end;
(4) Discard the reads without adaptor and inserted fragment at the 3'-end;
(5) Trim the adapter sequence at the 3'-end;
(6) Discard the reads whose length are less than 18nt after trimming.

Experiment IP Input/Mock Fragment_length Count_of_narrow_peak
ulk3_ChIP IP_Ulk3 Input 192(preset) 1470
shCtl_3AChIP IP3A_shCtl Inp_shCtl 199(preset) 67627
shulk3_3AChIP IP3A_shULK3 Inp_shulk3 197(preset) 2020

Software

Raw data files from ChIP-seq assays were aligned to the GRCh38 genome with Bowtie2 Version 2.3.0 (http://bowtie-bio.sourceforge.net/bowtie2). Duplicates were removed with Picard (https://broadinstitute.github.io/picard/) and, for peak detection, MACS2 software (http://liulab.dfci.harvard.edu/MACS) was used with a p-value cutoff of 1.00e–04. Peaks were annotated with HOMER (http://homer.ucsd.edu/homer/index.html). The Integrative Genomics Viewer (http:// software.broadinstitute.org/software/igv/) was used for graphic illustration of ChIP-seq peaks.
